# Decreasing Ambulatory CLABSIs in Oncology Patients

**DOI:** 10.1097/pq9.0000000000000698

**Published:** 2024-02-21

**Authors:** Angie Blackwell, Brittney K. Anderson

**Affiliations:** From the * Hematology/Oncology Unit, Clinical Quality Department, Children’s Hospital of Orange County, California, USA; † Clinical Quality Department, Children’s Hospital of Orange County, California, USA

## Background:

Central line–associated bloodstream infections (CLABSIs) pose a significant and costly risk associated with central line placement. In the United States, more than 200,000 preventable CLABSIs occur annually, resulting in substantial financial burdens. Recent changes in reimbursement policies have incentivized efforts to reduce CLABSI rates, primarily within hospital settings. However, limited understanding exists regarding the epidemiology of these infections in the ambulatory setting of pediatric oncology. Potentially life-threatening ambulatory CLABSIs (aCLABSIs) have serious implications, including increased hospitalizations, medication needs, line removal potential, reduced patient satisfaction, and poor outcomes.^1^ The literature suggests that analyzing pathogen distribution compared to hospital-acquired CLABSIs can offer insights into their causes and inform prevention strategies.^2^

## Methods:

**Fig. 1.**
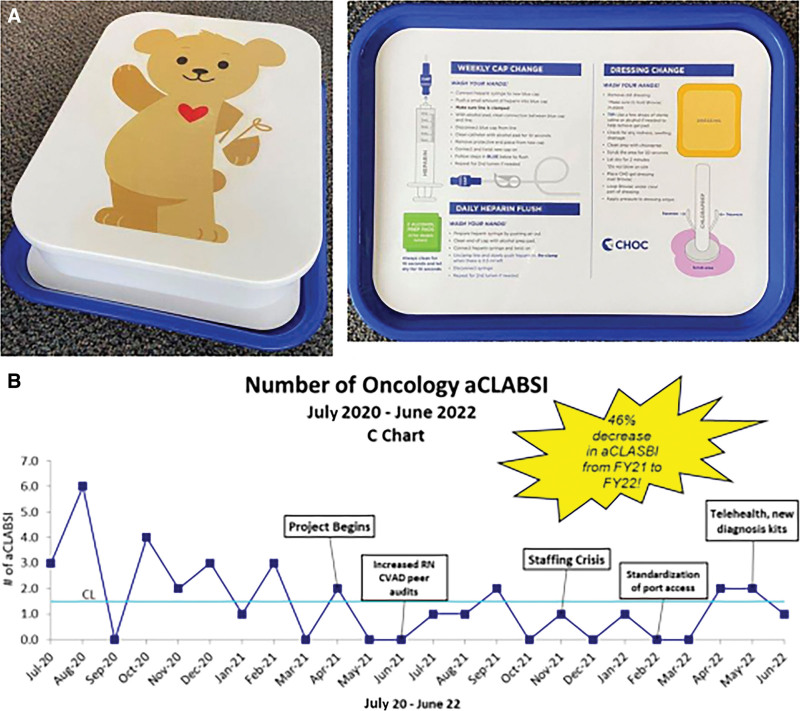
A, Home kit: storage box and tray liner step-by-step. B, C chart with plan-do-study-act cycle annotations.

Using improvement science methodology, we identified factors contributing to aCLABSIs within our institution. We constructed a driver diagram around key themes of family education, home hygiene, and standardized central venous access device care across settings. Based on these key drivers, we implemented several tests of change using plan-do-study-act methodology with the goal of decreasing aCLABSIs at our institution. The first improvement cycle introduced a home kit (Fig. 1A) for newly diagnosed families. The kit features a cafeteria-style tray creating a dedicated, clean surface for setting up the line care procedure. The tray includes a laminated insert with clear, concise line care instructions available in English and Spanish. Our second test of change focused on reinforcing education and alleviating caregiver apprehension with line care following the initial discharge home. To achieve this, we implemented a novel approach that leverages telehealth technology. Within 1–2 days following the initial discharge home, nursing staff conduct a telehealth session with the patient and their caregivers. During the telehealth session, nursing staff virtually observe the families as they perform line care procedures in the comfort of their own homes. They provide real-time guidance, answer questions, and offer reassurance. The third cycle optimized standardized central venous access device kits for outpatient areas to minimize waste. Peer audits were conducted to monitor compliance with the newly designed kits and verify that healthcare providers were following established best practices.

## Results:

Implementation of these plan-do-study-act cycles yielded a remarkable 58.3% reduction in aCLABSIs (Fig. 1B). This outcome underscores the impact of improvement science methodologies on patient safety and care quality.

## Conclusions:

This project demonstrates the power of improvement science tools to effect meaningful changes in patient safety practices. The successful outcomes are transferable to other healthcare organizations facing similar challenges. The project emphasizes the value of tailored interventions, interdisciplinary collaboration, data-driven decision-making, and a culture of continuous improvement. As institutions adopt these lessons, they can enhance patient safety and care outcomes, promoting better quality healthcare.
